# Molecular Approaches to Genetically Improve the Accumulation of Health-Promoting Secondary Metabolites in Staple Crops—A Case Study: The *Lipoxygenase-B1* Genes and Regulation of the Carotenoid Content in Pasta Products

**DOI:** 10.3390/ijms17071177

**Published:** 2016-07-21

**Authors:** Grazia M. Borrelli, Daniela Trono

**Affiliations:** Consiglio per la Ricerca in Agricoltura e l’Analisi dell’Economia Agraria, Centro di Ricerca per la Cerealicoltura, S.S. 673 Km 25,200, 71122 Foggia, Italy; graziamaria.borrelli@crea.gov.it

**Keywords:** phytochemicals, human health, metabolic pathway, metabolite analysis, enzymatic activity, transcript profile, candidate gene, biotechnological breeding, lipoxygenase, pasta-products

## Abstract

Secondary metabolites, also known as phytochemicals, represent a large subset of plant molecules that include compounds with health-promoting effects. Indeed, a number of epidemiological studies have shown that, when taken regularly and in adequate amounts, these molecules can have long-term beneficial effects on human health, through reduction of the incidence of degenerative diseases, such as cardiovascular diseases, obesity, diabetes, and cancer. As the dietary intake of these phytochemicals is often inadequate, various strategies are in use to improve their content in staple crops, and the end-products thereof. One of the most effective strategies is crop improvement through genetic approaches, as this is the only way to generate new cultivars in which the high accumulation of a given phytochemical is stably fixed. Efforts to genetically improve quality traits are rapidly evolving, from classical breeding to molecular-assisted approaches; these require sound understanding of the molecular bases underlying the traits, to identify the genes/alleles that control them. This can be achieved through global analysis of the metabolic pathway responsible for phytochemical accumulation, to identify the link between phytochemical content and the activities of key enzymes that regulate the metabolic pathway, and between the key enzymes and their encoding genes/alleles. Once these have been identified, they can be used as markers for selection of new improved genotypes through biotechnological approaches. This review provides an overview of the major health-promoting properties shown to be associated with the dietary intake of phytochemicals, and describes how molecular approaches provide means for improving the health quality of edible crops. Finally, a case study is illustrated, of the identification in durum wheat of the *Lipoxygenase-B1* genes that control the final carotenoid content in semolina-based foods, such as pasta products.

## 1. Introduction

The secondary metabolites from plants, which are known as phytochemicals, represent a large subset of plant chemicals, but their importance in both plant metabolism and human health has only been recognized more recently. This is because, unlike the primary metabolites, carbohydrates, lipids and proteins, which are ubiquitously present at high concentrations in all living organisms, in which they share the same essential functions (i.e., growth, development and reproduction), secondary metabolites are present at much lower concentrations, are restricted to specific groups of organisms and are produced in response to particular conditions [1]. Likewise, in human nutrition attention has been focused for years on the energy demand that needs to be satisfied through the correct combination of carbohydrates, lipids, and proteins in the diet, whereas the impact of secondary metabolites on human health has been ignored for a long time.

In the last decades, evidence has accumulated of the direct involvement of secondary metabolites in various processes related to plant fitness, such as defense against biotic and abiotic stress, formation of symbiosis at the root level, and reproductive processes through attraction of animals involved in pollination or seed dispersal [2]. In the same manner, the recognition of the biological properties of a number of secondary metabolites has led to the revaluation of these compounds also from the health point of view. Although phytochemicals do not have roles as nutrients, increasing epidemiological evidence has associated the consumption of foods rich in phytochemicals, such as fruit, vegetables, and grains to reduced incidence of cardiovascular diseases (CVD), obesity, diabetes, cancer, and other chronic degenerative diseases [3].

Nowadays, the dietary intake of bioactive compounds is often inadequate, and worldwide, people are becoming even more susceptible to the development of degenerative diseases [4]. Therefore, the demand for new effective strategies that are aimed at prevention of these diseases has increased considerably in the last few decades. One approach is to increase the content of health-promoting compounds in staple crops and their end products. This can be effectively achieved by genetic improvement of crops through proper breeding programs, which represent the only certain way to generate new cultivars in which a desirable phytochemical content is stably fixed.

From the late 19th century, plant breeding relied on phenotypic selection. Thus the improvement of a quality trait, such as the accumulation of a desirable compound, was achieved through screening of hundreds, or even thousands, of plants. Laboratory tests were then used to evaluate the phytochemical content and to select the plants of superior quality. The entire process was relatively long (>10 years) and laborious. Fortunately, over the years, with the advent of molecular genetics, crop improvement has rapidly evolved from classical breeding approaches to biotechnological breeding approaches, such as marker-assisted selection (MAS) [5], genetic transformation [6], and very recently, genome editing [7]. These advanced technologies require sound understanding of the molecular bases underlying the accumulation of a bioactive compound in a given crop, to be able to identify the candidate gene that controls its levels. The challenge is then to introduce this candidate gene into an elite cultivar, while retaining its performance attributes.

Two approaches can be used to identify and validate such a candidate gene: (i) the positional approach; and (ii) the functional approach [8]. The positional approach is based on the identification of a potential candidate gene among the genes that map around the quantitative trait locus (QTL) that is associated with the trait. This is followed by its validation through the demonstration that polymorphic variants of the candidate gene (i.e., alleles) can explain the variability of the trait. Conversely, the functional approach bypasses the QTL step, as it relies on identification of the candidate gene through its functional relation to the trait, and in particular through the association between its allelic variation and the variation in the flux rate through the pathway that is responsible for the metabolite accumulation. The metabolic pathway is often a multi-step event that involves different enzyme-catalyzed reactions, although the rate of the overall pathway is generally regulated by the rate of one, or a few, reactions that are catalyzed by the so-called “rate-limiting” enzymes. Allelic variation at the gene that encodes a key regulatory enzyme of the pathway might have the potential to modify the functioning of the enzyme, and consequently, the accumulation of the desired phytochemical. Therefore, the combination of target metabolite determination with enzymatic assays as well as gene-sequence and expression analyses might represent a valuable multi-disciplinary approach for the identification of favorable alleles that, when used as markers, can help a breeder to successfully achieve the goal of biofortification of staple crops.

Here, we provide an overview of the main classes of plant phytochemicals, and we summarize the current knowledge of their health-promoting effects. Then, we focus on the molecular strategies and tools that can be applied to effectively manipulate the phytochemical levels in agronomically important crops. Finally, we describe a functional study of the *lipoxygenase-B1* gene family in durum wheat that controls the final carotenoid content in pasta products.

## 2. General Overview of Plant Secondary Metabolites

**Figure 1:**
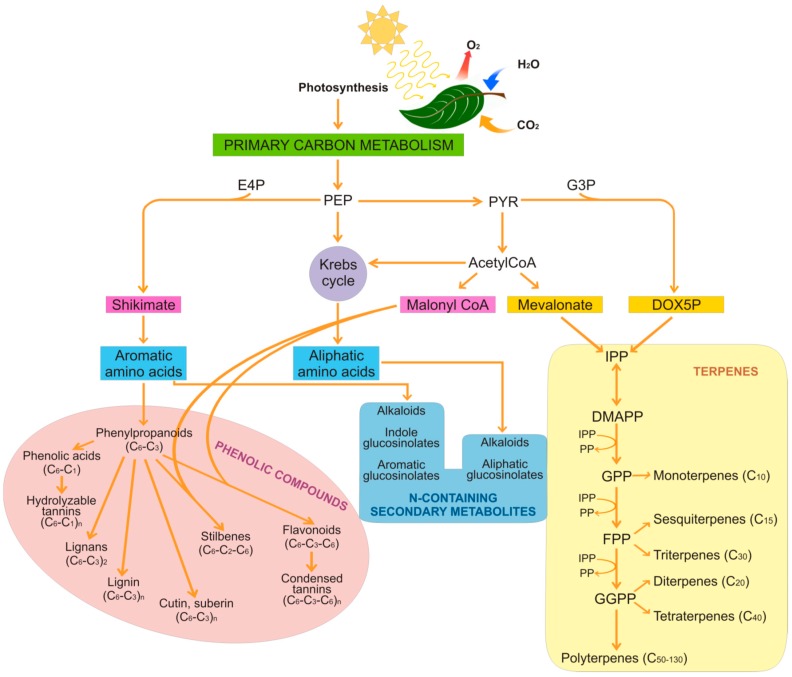
Schematic representation of the biosynthesis of secondary metabolites. E4P, erythrose 4-phosphate; G3P, glyceraldehyde 3-phosphate; PEP, phosphoenolpyruvate; PYR, pyruvate; DOX5P, deoxyxylulose 5-phosphate; IPP, isopentenyl pyrophosphate; DMAPP, dimethylallyl pyrophosphate; PP, pyrophosphate; GPP, geranyl pyrophosphate; FPP, farnesyl pyrophosphate; GGPP, geranylgeranyl pyrophosphate.

Secondary metabolites can essentially be divided into three main groups that are based on their structures and the metabolic pathways responsible for their biosynthesis (Figure 1): phenolic compounds; terpenoids; and nitrogen-containing compounds, i.e., alkaloids and glucosinolates [9].

### 2.1. Phenolic Compounds

Phenolic compounds represent one of the largest groups of secondary metabolites produced in plants (i.e., >8000 different structures), and particularly in edible plants (e.g., vegetables, fruit, cereals). These include several structurally different compounds that have at least one aromatic hydroxyl-substituted ring in common. Phenolic compounds are produced primarily via the shikimate pathway that gives rise to phenylpropanoids (Figure 1).

The combination of the shikimate pathway with the mevalonate pathway leads to the generation of the flavonoids [10]. Condensation and polymerization reactions produce hydrolysable and condensed tannins, lignans, lignin, cutin, and suberin. Phenolic compounds are involved in a number of physiological processes in plants, such as pollination, resistance to pathogens and predators, and resistance to abiotic stress [10]. Although they can exist in their free forms, phenolic compounds are commonly conjugated to sugars or proteins, and can also occur as esters and methyl esters, probably because most of them are toxic compounds that are at least in part neutralized in their bound form. Due to the large number and heterogeneity of structures, phenolic compounds can be classified in different ways. On the basis of their carbon chain, they can be distinguished in different classes that range from simple phenols to highly polymerized compounds (Table 1).

**Figure 2:**
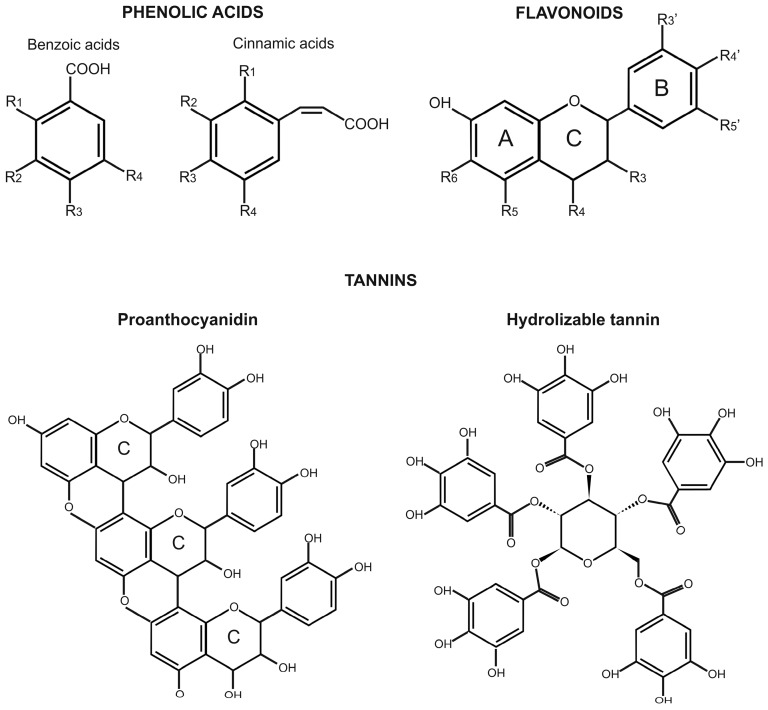
General structures of the three most important classes of phenolic compounds for human health. Benzoic acids: *p*-hydroxybenzoic acid, R_3_=OH; vanillic acid, R_2_=OCH_3_ and R_3_=OH; gallic acid, R_2_=R_3_=R_4_=OH; syringic acid, R_2_=R_4_=OCH_3_ and R_3_=OH. Cinnamic acids: *p*-coumaric acid, R_3_=OH; caffeic acid, R_3_=R_4_=OH; ferulic acid, R_2_=OCH_3_ and R_3_=OH; sinapic acid, R_2_=R_4_=OCH_3_ and R_3_=OH. Flavonoids: R_3_–R_6_=H or other groups such as OH or oxo- groups. For most food flavonoids: R_4’_=R_6_=H and R_5_=OH. In biochanin A, R_4’_=OCH_3_; formononetin, R_4’_= OCH_3_ and R_5_=R_6_=H; glycetein, R_5_=H and R_6_=OH.

The three most important classes of phenolic compounds for human health are phenolic acids, flavonoids, and tannins. Phenolic acids are the simplest phenolic compounds found in nature, and they account for one-third of all of the phenolic compounds in the human diet. Phenolic acids include two major groups, benzoic acids and cinnamic acids, which have seven (C_6_–C_1_) and nine (C_6_–C_3_) carbon atoms, respectively (Figure 2). These compounds are characterized by an aromatic ring, a carboxylic group, and one or more hydroxyl and/or methoxyl groups. Cinnamic acids are more commonly found in nature than benzoic acids. Among the cinnamic acids, caffeic acid and its esterified derivatives are the most abundant in fruit, whereas ferulic acid and its derivatives are the most abundant in cereal grains [11].

Flavonoids are the most widely distributed phenolic compounds in plants, as they account for more than half of the 8000 phenolic compounds found in nature [12]. These occur in various tissues and organs of edible crops, such as fruit, leaves, roots, and tubers, as well as in legumes, tea, coffee, herbs, and spices. Flavonoids have a common structure that consists of two aromatic rings (the A and B rings), joined by a C_3_ chain that is generally in the form of a heterocyclic ring (the C ring) (Figure 2). Aromatic ring A comes from the malonate pathway, whereas ring B comes from the shikimate pathway. Substitutions to ring C result in different types of flavonoids, such as flavones, flavonols, flavanones, flavanols, anthocyanidins, and isoflavones, whereas substitutions to rings A and B give rise to different compounds within each type of flavonoid.

Tannins are the third most important group of phenolic compounds. These include a large number of oligomers and polymers that can form complexes with starch, proteins, cellulose, and minerals. Tannins can be distinguished as two groups: hydrolysable tannins and condensed tannins, which are also known as proanthocyanidins. Hydrolysable tannins are compounds formed from gallic acid or epigallic acid units that are condensed to a central sugar molecule, while condensed tannins are polymers of catechin and epicatechin, or similar units (Figure 2). Proanthocyanidins are more widely distributed in edible plants than hydrolysable tannins, as they are widespread in fruit, legumes, nuts, and minor cereals, such as sorghum and barley, and they tend to concentrate in the peel of fruit and the bran of grains.

Phenolic compounds are considered to be powerful antioxidants due to the reactivity of their phenol moiety. Their main mode of action as antioxidants is radical scavenging via hydrogen atoms or electron donation, together with the delocalization of the unpaired electron within the aromatic ring [13]. Substituents on the aromatic rings affect their stabilization, and therefore affect the radical-quenching of these phenolic compounds. Of note, flavonoids are among the most powerful antioxidants that have been obtained from plants. Their strong antioxidant capacity is due to the presence of hydroxyl groups in positions 3’ and 4’ of ring B, which participate in the electron delocalization and stabilize the radical that is formed, and of a double bond between the C2 and C3 carbons in ring C, together with a carbonyl group at the C4 carbon, which make delocalization of an electron from ring B possible [14].

Another class of compounds that contains a phenol ring is represented by the alkylresorcinols. However, compared to phenolic compounds, alkylresorcinols represent a very distinct group, as they have both polar and nonpolar properties. Indeed, in addition to the phenol ring with two hydroxyl groups, alkylresorcinols also have a hydrophobic and mostly saturated alkyl chain. The rich sources of alkylresorcinols include cereal grains (i.e., from wheat, rye, triticale, barley), in which these compounds accumulate in the cuticle layer. There is evidence that alkylresorcinols protect cellular lipids from oxidative damage [15].

### 2.2. Terpenes

Terpenes and terpenoids (i.e., terpene-like compounds) are lipid-soluble compounds that represent the most abundant and structurally diverse group of plant secondary metabolites (with >50,000 different structures) [16]. These are normally produced in vegetative tissues and flowers, and occasionally in roots [17]. They are also referred to as isoprenoids, as they are all derived from the repetitive fusion of isoprene (C_5_H_8_) units. All isoprenoids are derived from the common precursor isopentenyl pyrophosphate (IPP), which is ubiquitously synthesized through the mevalonate pathway or the deoxyxylulose-5-phosphate pathway [18] (Figure 1). IPP isomerizes to dimethylallyl pyrophosphate, and the condensation between IPP and dimethylallyl pyrophosphate gives rise to geranyl pyrophosphate, the precursor of all of the monoterpenes. The IPP additions that follow generate other prenyl diphosphates, which are the precursors of all of the other terpenes. In plants, the synthesis of monoterpenes (C10), sesquiterpenes (C15), diterpenes (C20), triterpenes (C30), tetraterpenes (C40), and polyterpenes (C50–C130) has been reported ([19], and references therein).

As shown in Table 2, terpenoids include compounds that are implicated in both primary and secondary metabolism. Gibberellin, cytokinins, abscisic acid, and brassinosteroids are hormones that are involved in plant growth and development, whereas carotenoids, and compounds that contain an isoprenyl side chain, such as chlorophyll, phylloquinone, plastoquinone, and tocopherols have functions in photosynthesis [16]. However, most of the terpenoids are secondary metabolites that have crucial roles as pollinator attractants, and in the defense of plants against herbivores and microbial pathogens [19].

**Figure 3:**
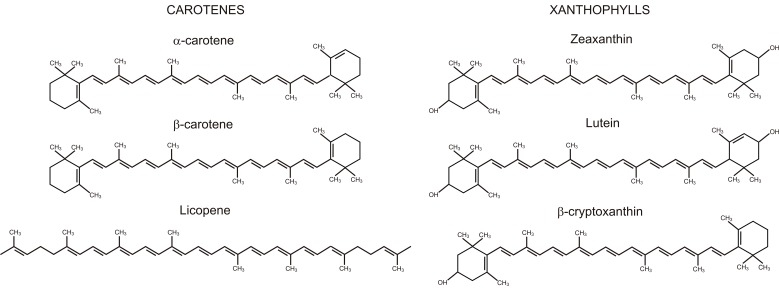
Structures of carotenes and xanthophylls that are important for human health.

The carotenoids are a class of terpenoids that have recognized beneficial functions for human health [20]. These are C40 isoprenoids, and they represent the second most abundant pigments in nature (with >750 compounds). There are two general classes of carotenoids: carotenes, which consist exclusively of hydrogen and carbon; and xanthophylls, which also contain oxygen (Figure 3). As animals and humans cannot synthesize carotenoids de novo, the diet is relied on for these essential compounds. Pro-vitamin A carotenoids, such as α-carotene, β-carotene, and β-cryptoxanthin (Figure 3), are important precursors of retinol (i.e., vitamin A), retinal, retinoid, and retinoic acid in animals and humans [20,21], whereas some xanthophylls, such as lutein and zeaxanthin (Figure 3), are essential components of macular pigments. In addition, carotenoids act as antioxidants. In particular, they can efficiently quench singlet oxygen. This implies energy transfer from the singlet oxygen to the carotenoid molecule, to yield ground-state oxygen and a triplet excited carotenoid molecule; the excited carotenoid can then return to the ground state by dissipating this energy as heat [22]. Chemical quenching of singlet oxygen has also been reported, which leads to oxidation of the carotenoid molecule [23].

Among the various radicals, carotenoids most efficiently react with peroxyl radicals generated during lipid peroxidation. For this reason, and because of their lipophilicity, carotenoids are believed to have important roles in the protection of cell membranes against oxidative damage [22]. The food sources of these compounds include various fruit and vegetables.

### 2.3. Nitrogen-Containing Compounds

Secondary metabolites that contain nitrogen include alkaloids and glucosinolates. Alkaloids represent a large class of nitrogen-containing compounds (with >12,000) where the nitrogen is derived from an amino acid, such as tyrosine, tryptophan, lysine, and aspartate (Figure 1). Compared to the other classes of secondary metabolites, alkaloids have great structural diversity, and for this reason, there is no single uniform classification for these compounds. Most of them are toxic to other organisms, and therefore they have important roles in plants in terms of their defense against pathogen and herbivore attack [24]. Once produced, alkaloids are stored in the cell vacuole and then released when mechanical damage breaks the vacuole. Alkaloids include a number of molecules of pharmacological interest, such as morphine, codeine, atropine, quinine, vincristine, and vinblastine, which have important therapeutic activities that include antibacterial, antiviral, antimalarial, antiproliferative, and antimetastatic effects in various types of cancers [25].

**Figure 4:**
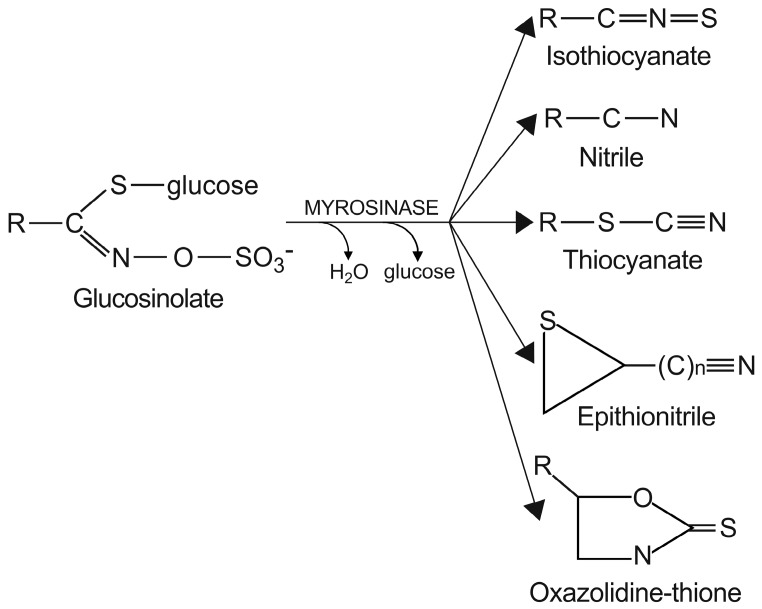
Hydrolysis of glucosinolates catalyzed by myrosinase. Myrosinase catalyzes the hydrolysis of the thioglucoside linkage, thus leading to the formation of different products. R represents the amino-acid-derived side-chain.

Conversely, there is a nutritional interest in the class of glucosinolates, which is a relatively small, but diverse, group of about 200 compounds. These are substituted esters of thio-amino acids that are mainly limited to species of the order of Brassicales, such as broccoli, cauliflower, cabbage, Brussels sprouts, red radish, horseradish, and mustard [26]. In plants, glucosinolates act as pesticides and as herbivore repellents [27]. Again, these compounds are stored in the cell vacuole, and upon cell disruption they are rapidly hydrolyzed by myrosinase and converted to thiocyanate, isothiocyanate, nitrile, epithionitrile, and oxazolidine-thione (Figure 4), which are toxic for pathogens and herbivores [28].

Based on the structure of the amino-acid precursor, glucosinolates can be defined as aliphatic, indole, and aromatic [29,30] (Table 3). Aliphatic glucosinolates are derived from alanine, leucine, isoleucine, methionine, and valine, while indole glucosinolates and aromatic glucosinolates are derived from tryptophan and phenylalanine or tyrosine, respectively. The presence of glucosinolates varies not only among plant species, organs, and tissues, but also under specific environmental conditions. Of note, nearly all of the potential health benefits of glucosinolates are due to their hydrolytic products and in particular to the isothiocyanates [31]. Mammalian tissues do not contain myrosinases, but degradation of glucosinolates still occurs in humans through the gut microflora. Indeed, several studies have identified gut bacterial strains that are associated with the formation of isothiocyanates from glucosinolates ([32], and references therein).

## 3. Health Benefits of Secondary Metabolites

Chronic diseases, such as CVD, diabetes, cancer, and neurodegenerative and age-related diseases are worldwide health problems that cause severe disability and even death. Epidemiological studies have shown that due to their significant content of phytochemicals, regular consumption of fruit, vegetables, and grains is strongly associated with reduced risk of these chronic diseases.

### 3.1. Cardiovascular Diseases

Cardiovascular diseases are the main cause of death and disability in developed countries. Epidemiological studies have shown that there is a strong inverse association between intake of some phytochemicals and incidence of hypertension, myocardial infarction, stroke, and atherosclerosis. In particular, consumption of foods rich in phenols has been shown to have a protective effect upon CVD. This is particularly convincing for flavanols from cocoa-derived products and tea, the consumption of which leads to reduced blood pressure and improved endothelial function [33,34]. Although conclusive data have yet to be realized, promising findings have also been obtained for the effects of resveratrol (from berries, grapes, plums, peanuts) on ischemic heart disease [35], of anthocyanins (from berries, grapes, citrus fruit) on low-density-lipoprotein cholesterol [36], and of quercetin (from onions, apples, berries, grapes, tea) on hypertension and atherosclerosis [37]. As for carotenoids, crocin (from saffron) has proven to be a good candidate for inhibition of platelet aggregation and for protection against the apoptosis of platelets induced by oxidative stress [38], while lycopene (from tomato) appears to improve endothelial function in patients with CVD, and to reduce incidence of coronary heart disease [39]. Finally, there is evidence of the involvement of the dietary intake of isothiocyanates (from *Brassica* species) in improved post-ischemic ventricular function, in reduced myocardial infarct size, and in decreased cardiomyocyte apoptosis in mammals [40]. Allicin (from garlic, onion, shallot, leek) is another organosulfur compound, and it has been shown to protect the cardiovascular system through induction of vasorelaxation, and to alleviate various pathological conditions of CVD [41].

### 3.2. Obesity

Obesity is associated with increased risk of CVD, type 2 diabetes, and gastrointestinal cancer. At the cellular level, obesity is characterized by increased number and size of adipocytes, which are differentiated from fibroblastic pre-adipocytes in adipose tissue. Evidence has been accumulated that the dietary intake of foods rich in phenols can prevent obesity by induction of pre-adipocyte and adipocyte apoptosis, and by inhibition of adipocyte lipid accumulation. In particular, catechins and epigallocatechin gallates (from green tea), resveratrol, and curcumin (from the curry spice turmeric) can reduce the viability of adipocytes and proliferation of pre-adipocytes, suppress adipocyte differentiation and triglyceride accumulation, stimulate lipolysis and fatty-acid β-oxidation, and reduce inflammation [42]. Moreover, epidemiological studies in humans have associated higher dietary intake and serum levels of carotenoids with decreased adiposity. This is probably due to the anti-adiposity and anti-inflammatory actions of these compounds and their derivatives. Indeed, cell and animal studies have demonstrated that some carotenoids, such as fucoxanthin-, astaxanthin-, and β-carotene-derived retinoids, can suppress adipogenesis and activate lipid oxidation and thermogenesis in mature adipocytes [43]. As for other classes of phytochemicals, animal studies have revealed that an onion extract has anti-obesity effects [44]. In the same study, sulfur-containing components, such as cycloalliin, *S*-methyl-l-cysteine, *S*-propyl-l-cysteine sulfoxide, dimethyl trisulfide, and especially *S*-methyl-l-cysteine sulfoxide, were reported to be effective for inhibition of the formation of oil droplets in cells, which suggests that these compounds are involved in the anti-obesity effects of the onion extract.

### 3.3. Diabetes

Phytochemicals prevent type 2 diabetes through different mechanisms. Experimental and clinical evidence has shown that dietary phenols modulate carbohydrate metabolism through the regulation of α-amylase and α-glucosidase activities. This thus protects pancreatic β-cell integrity and physiology against glucose toxicity, reduces postprandial glycemic levels, and enhances insulin releasing activity [45,46,47]. Due to the high content of ferulic acid, flavonoids, and alkylresorcinols in cereal wholegrain foods, these have been demonstrated to delay or even prevent the development of type 2 diabetes and its complications [48]. Fruit, such as berries, grapes, and pomegranates, vegetables such as artichoke and common bean, and popular drinks like cocoa, coffee, and green tea, are all rich in phenolic compounds, and these have also been shown to have beneficial antidiabetic effects [46,49,50,51]. For the other phytochemicals, there is evidence that organo-sulfur compounds in onion extracts can decrease lipid levels and increase glucose tolerance [44]. Finally, an association between the intake of foods rich in α-carotene and β-carotene and reduced risk of diabetes 2 has been reported recently [52].

### 3.4. Cancer

Dietary phytochemicals can have complementary and overlapping protective mechanisms of action against cancers. As for phenolic compounds, phytochemicals have protective effects mainly by acting against UV-radiation-induced inflammation, oxidative stress, DNA damage, and suppression of immune responses. Catechin and proanthocyanidins (from grape seeds, cocoa, apples, peanuts, almonds, cranberries, blueberries) have been shown to be particularly good for protection of the skin from the adverse effects of the UV radiation that causes skin cancers [53]. Anthocyanidins (from berries, cherries) and genistein (from soy) can have anticancer activities through down-regulation of the expression of cancer-related genes [54,55]. Potential beneficial effects of quercetin (from onions, apples, tea) in adjuvant chemotherapy in prostate, breast, colon, and lung cancers have been demonstrated both in vitro and in vivo [56]. Numerous preclinical findings based on human and animal studies have also suggested that resveratrol is a promising tool for cancer prevention and treatment, through its antioxidant activity [57]. As for carotenoids, there is evidence that a diet rich in β-carotene, lycopene, lutein, and zeaxanthin can prevent the incidence of colorectal cancer at an early stage of disease progression [58]. Finally, epidemiological studies have suggested that isothiocyanates are protective against cancers of the lung and alimentary tract [59].

### 3.5. Alzheimer’s Disease

Neurodegenerative disorders, and especially Alzheimer’s disease (AD), represent a common cause of cognitive decline and dementia, particularly in older people. There is evidence that the dietary intake of foods rich in flavonoids, such as wine, tea, and chocolate, are associated with improved performance for several cognitive abilities [60,61]. Animal studies have demonstrated that at the cellular level, phenolic compounds, such as ferulic acid, myricetin, nordihydroguaiaretic acid, and rosmarinic acid, can prevent the development of AD pathology through reduction of the aggregation of amyloid-β protein in the brain parenchyma, which is responsible for the loss of synapses and impairment of neuronal functions [62]. In addition, other studies have suggested that the protective effects of some phenolic compounds against neurodegenerative diseases, such as quercetin, curcumin, resveratrol, and naringenin, are related to their inhibition of acetylcholine esterase activity, which enhances cholinergic transmission [63]. In vitro and in vivo studies have demonstrated that similar to phenolic compounds, β-carotene, lycopene, retinol, and retinoic acid can also prevent AD symptoms, primarily through inhibition of amyloid fibril formation [64,65,66].

### 3.6. Eye Diseases

Lutein and zeaxanthin constitute the main pigments in the yellow spot of the human retina, which protect the macula from damage by blue light and harmful reactive oxygen species. Lutein and zeaxanthin are the most common xanthophylls in green leafy vegetables (e.g., broccoli, spinach, collard green, kale, corn, orange pepper, zucchini, and squash), fruit (e.g., kiwi, grapes), and egg yolk, although these are also found at relatively high levels in maize, einkorn, and durum wheat grains and their food products. In the last decade, several epidemiological and clinical studies have demonstrated that consumption of such foods is related to reduced risk of two major age-related eye diseases: macular degeneration and cataracts [67]. β-carotene is abundant in vegetables (e.g., carrots, asparagus, sweet potatoes, spinach, kale, pumpkin, squash, peppers, oregano, paprika, parsley) and fruit (e.g., cantaloupes, grapes, plums, apricots), and after its ingestion, it is converted into retinol, or vitamin A, which serves as the precursor of the light sensor molecules in the retina. An adequate intake of β-carotene can prevent degenerative eye damage, such as night blindness, xerophthalmia, Bitot’s spot, keratitis, and keratomalacia [68].

## 4. Molecular Tools for the Identification of Candidate Genes

A possible strategy to improve the content of a desired phytochemical in an agronomically relevant crop is based on the knowledge of the genes that control its accumulation. Through proper biotechnological breeding approaches, these genes can be used as tools for the selection of new high-quality genotypes in which the trait is stably fixed. Candidate gene can be identified through the general knowledge of the biochemical and molecular regulation that governs the metabolic pathway responsible for accumulation of the desired phytochemical, together with an understanding of the mechanisms that underlie the individual enzymatic reactions that make up the pathway. Indeed, the rationale for function-dependent strategy states that metabolite accumulation represents the outcome of the functioning of key enzymes that catalyze the rate-limiting reactions of the pathway and that regulate its flux rate. Differences in the functioning of a key enzyme can be related to differences in the amount of the enzyme and/or to different variants of the enzyme (known as allozymes), which are characterized by differences in their amino-acid sequences, and consequently, in their kinetic behavior. This is related, in turn, to the presence of different alleles of the gene that encode the key enzyme, which differ from each other in terms of polymorphisms in their regulatory and/or coding regions, such as insertions/deletions (Indels) or single nucleotide polymorphisms (SNPs). A favorable allele that is associated to a more active/efficient allozyme in a biosynthetic pathway or to a less active/efficient allozyme in a catalytic pathway, might represent a valuable candidate that is associated to greater accumulation of a desired phytochemical, and thus should be useful in the selection of new, biofortified genotypes.

**Figure 5:**
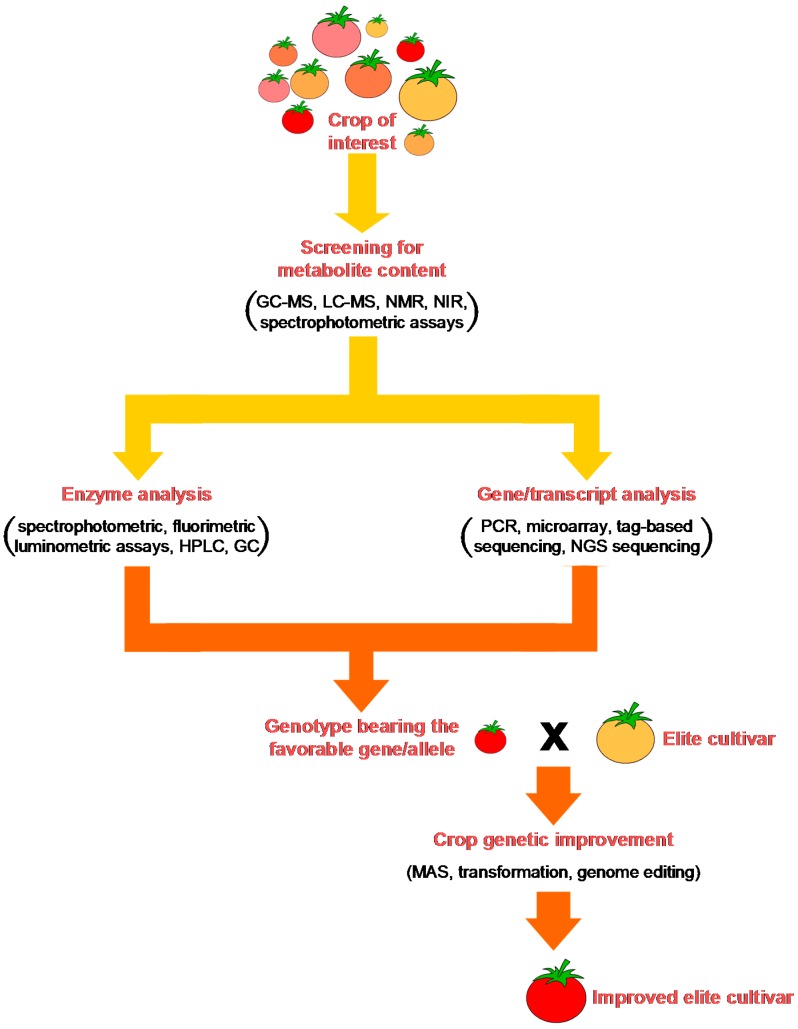
Outline of how a functional approach can contribute to identification of a candidate gene associated with high levels of a health-promoting phytochemical in a staple crop. A germplasm collection is screened to determine the variability in nature of the trait. Genotypes characterized by contrasting metabolite accumulation are subjected to enzymatic analysis to identify key enzyme variants (i.e., allozymes) that are characterized by contrasting activities/efficiencies, and molecular analysis to identify alleles that encode the different allozymes and to evaluate their expression levels. Then, on the basis of both the enzymatic and molecular information, a candidate gene/allele can be identified that can then be efficiently used in advanced breeding programs that are aimed at the introgression of the trait of interest into an elite cultivar.

In the light of this, it is evident that an understanding of all of the aspects of a metabolic pathway requires multidisciplinary expertise, which includes: (i) analytical skills for identification of the molecules of interest, their precursors, and their degradation products; (ii) biochemical skills for characterization of the enzymes, understanding of their biochemical properties, and identification of allozymes with the desirable performance; (iii) molecular skills for identification and characterization of their encoding genes/alleles and understanding how their expression is regulated. A possible multidisciplinary approach to enhance the production of secondary metabolites in crops is outlined in Figure 5.

Nowadays, the functional approach for identification of candidate genes/alleles can be advanced by taking advantage of publicly available databases and repositories. In particular, there are databases that contain metabolite (ChEBI, GMD, KEGG compound, PubChem) [69,70,71,72], reaction (BRENDA, ExPASy-enzyme, KEGG enzyme/KEGG reaction, Rhea BioPAX, Sabio-RK) [73,74,75,76,77] and plant-specific metabolic pathways (e.g., KEGG pathway, MetaCrop, MetaCyc, PANTHER pathway, PlantCyc, Reactome) [78,79,80,81,82,83] that are available and under continuous updates [84]. In addition, with the recently emerged cheap and immense next-generation sequencing (NGS) technologies, rapid whole transcriptome and genome sequencing that was previously restricted to model plants has been extended to an increasing number of crops. Consequently, plant transcriptome-based databases are available (e.g., PlantransDB, PLEXdb) [85,86] that provide relevant information about genes expressed in certain plant tissues or organs at any given growth stage and under any particular conditions. As for plant genomes, there are a number of sequencing projects where cultivated species and their wild relatives have been completely sequenced, or are being assembled or are in progress (e.g., leuks.cgi, PlantGDB, Phytozome, and other species-specific databases) [87,88,89,90]. All of this heterogeneous information can now be integrated using in-silico approaches to establish a ranking of candidate genes on the basis of their relevance in a given pathway in the species of interest, and/or in closely related species. From these genes, the most promising can be selected for experimental validation through biochemical and molecular approaches.

### 4.1. Metabolite Analysis

To choose the breeding strategy that is more adapted for improvement of the phytochemical content in a crop of interest, a metabolite analysis has to be performed that determines the natural variability of the trait in the genotypes of a large germplasm collection. To date, the most frequently used approach in plant breeding has been based on targeted analyses that have focused on a restricted group of metabolites. In contrast, the broad, untargeted approaches that allow simultaneous determination of hundreds of compounds is still poorly applied for plant breeding purposes.

Metabolite analyses can be performed using different techniques, such as gas chromatography (GC), liquid chromatography (LC), also as high performance (HPLC) and ultra performance, capillary electrophoresis, near-infrared (NIR) spectroscopy, and nuclear magnetic resonance (NMR). In plant metabolite analyses, GC and LC are often interfaced with mass spectrometry (MS) as the detector, as the most used system at present. Both GC-MS and LC-MS involve separation of metabolites using a chromatographic approach followed by mass separation by MS. For the reproducibility, ease of use, and sensitivity in the nanomolar to picomolar range, GC-MS represents the most widely used technique for determination of plant metabolites [84]. As metabolite volatility is absolutely required for GC, this is the technique of choice for the determination of volatile compounds. GC is also widely used for determination of nonvolatile metabolites, including plant secondary metabolites such as terpenoids and phenolic compounds, which have to be chemically derivatized to become volatile [84]. LC-MS represents a valuable complement to GC-MS. LC is not influenced by volatility and thermal stability of the samples, and combined with MS it can detect different plant secondary metabolites, such as terpenoids, phenolic compounds, glucosinolates, and alkaloids, with a sensitivity in the picomolar to femtomolar range when it is used in combination with electron spray ionization MS. LC-based separations use two different approaches: C18 reverse-phase separation of hydrophobic metabolites; and hydrophilic interaction chromatography separation of hydrophilic metabolites [91]. To improve the separation of complex samples, different parameters can be varied, such as column chemistry, and programmed temperature method for GC and elution method for LC. Recently, an improvement to chromatographic separation was achieved with the use of multi-dimensional separation systems, such as two-dimensional GC (GC × GC) [92] and two-dimensional LC (LC × LC) [93]. These are based on the use of columns with different chemistries which minimize analyte co-elution and peak overlap in complex samples.

In both GC-MS and LC-MS, the eluent from the chromatographic step is transferred into an ion source where the dissolved metabolites are converted into ions. Different ionization techniques are available, with electron ionization as the most widely used in GC-MS, while the most common technique in LC-MS is electron spray ionization [94]. The mass spectrometer determines the size of the ionized molecule through the mass-to-charge ratio of each detected ion, as well as the number of the individual ions detected. The mass-to-charge ratio is used to identify the metabolite, whereas its quantification is obtained through the number of ions detected, which can then be related back to the metabolite concentration in the plant extract [94]. An additional advantage in MS is that precursor ions of particular mass-to-charge ratios obtained after the first round of MS can be fragmented in a collision gas cell, and the resulting daughter ions can then be separated and detected in a subsequent round of MS. This can be repeated one (MS/MS) or more (MS^*n*^) times, with the advantage of greatly enhanced specificity of the analysis.

Unlike the chromatographic techniques, NMR spectroscopy is a nondestructive technique that requires minimal sample preparation and no separation processes before metabolite detection. In NMR analysis, samples in deuterated water are placed in a magnetic field and irradiated with a radio frequency. The energy absorbed causes the transition of the hydrogen nuclei from a low-energy to a high-energy state. The subsequent emission of radiation due to the relaxation of the nucleus generates the resonance frequencies or signals, which are recorded and processed to yield an NMR spectrum. This can be related to the molecule structure, whereas the signal intensity is related to the molar concentration of the molecule [95]. Although time-saving, the use of NMR spectroscopy has some limitations. In particular, this technique requires large amounts of sample, its sensitivity is low (in the micromolar range), and the analysis of complex samples is difficult to achieve, as multiple signals from different metabolites frequently overlap, thus making their identification difficult.

Compared to chromatographic and NMR techniques, NIR spectroscopy is more rapid and cost effective. NIR is a vibrational spectroscopy that uses radiation in the 750 nm to 2500 nm wavelength range to obtain absorption spectra. The qualitative and quantitative information are then acquired through correct instrument calibration. Although NIR spectroscopy is not as sensitive as the chromatographic techniques, this simple method allows rapid, nondestructive analysis of high numbers of samples, and it is thus particularly suitable for plant breeding purposes [96].

Spectrophotometric assays have also been developed to determine the total content of given classes of phytochemicals ([97], and references therein). Although these approaches are much less specific and sensitive than the analytical methods described above, spectrophotometric assays provide a rapid, simple, and inexpensive tool for large-scale screening associated to plant breeding programs. After the extraction of the metabolites using the relevant solvent (e.g., ethanol, methanol, *n*-butanol, acetone), the assays are carried out spectrophotometrically at a wavelength specific for a given class of phytochemicals. Here, total phenolics can be assayed at 725 nm, total anthocyanins at 535 nm, total flavonols at 374 nm, total proantocyanidins at 500 nm, and total carotenoids at 450 nm.

### 4.2. Enzyme Analysis

Genotypes with contrasting metabolite contents can be analyzed with respect to the key regulatory enzymes of a pathway. This approach determines their catalytic properties and whether the differences observed in metabolite accumulation are related to the presence in these genotypes of enzymes with contrasting behaviors that differentially influence the flux rate of the pathway. Differences can be observed at two different levels: in the amount of the enzyme, and in its catalytic properties. Differences in the amount of an enzyme are the consequence of differences in the expression of its encoding gene, which, in turn, can be related to the existence of different alleles that are polymorphic in their regulatory regions. Conversely, differences in the catalytic properties are due to the existence of different allozymes, which are encoded by alleles that are polymorphic in their coding regions and that, for this reason, differ in their structure and function. All of the catalytic properties of an enzyme can be assessed through enzymatic assays.

The aim of any enzymatic assay is to follow the time-dependent formation of a product or the consumption of a reagent of the catalyzed reaction. In the simplest case, an enzyme reaction can be observed using a spectrophotometer to monitor the appearance or disappearance of a compound that absorbs in the UV/visible range. Other optical approaches include fluorimetric assays, which are more sensitive than absorbance assays, but which have the disadvantage that only a few compounds emit fluorescence, such as NADH and some artificial substrate analogs [98]. In addition, whereas for spectrophotometric assays the substrate or product concentration can be directly calculated from the absorbance signal through the Lambert-Beer law, in fluorimetric assays calibration curves at different substrate or product concentrations are strictly required. Turbidimetry can also be used to follow the enzymatic degradation of particles [99], while luminometry can be applied to monitor ATP-dependent reactions [100,101]. Enzymatic reactions without any optically active substrate or product cannot be followed directly by means of spectrophotometric of fluorimetric measurements. To render these enzymatic reactions detectable, an enzyme-coupled assay must be carried out. This consists in further converting a reaction product by a second enzyme to form a second product, and so on, until an optically active product is generated that thus produces a detectable signal. As the reaction rate measured must correspond to the rate of the first reaction, the coupled reactions should never become limiting; to achieve this, the enzymes for the coupled reactions should be added in excess compared to the first enzyme to be assayed [98].

The methods described above allow continuous, time-dependent monitoring of enzymatic reactions, which is a fundamental prerequisite for the correct determination of reaction rates. At the beginning, the signal varies linearly with varying substrate or product concentrations; then, the progress curve deviates from this linearity. Only the linear part of the progress curve should be taken into consideration for the determination of the reaction rate, which is calculated from the slope of the linear part of the progress curve, and is expressed as the amount of substrate consumed or product formed in a given time interval. Alternatively, there are also discontinuous assays (referred to as ‘end-point’ assays), in which aliquots of the reaction mixture are removed at various times and substrate depletion or product formation is determined by analytical techniques, such as HPLC or GC [102]. To avoid underestimation of the reaction rate, the time points selected for the determinations must fall within the linear portion of the progress curve of the enzymatic assay.

According to the Michaelis-Menten equation, the rate of an enzymatic reaction is hyperbolically related to the substrate concentration [103]. The binding of a substrate to the active sites of an enzyme occurs more efficiently at low concentrations, while with progressive occupation of the active sites, increasing substrate concentrations are required. When all of the active sites are occupied, the enzyme is saturated and reaches its maximal activity (*V*_max_), such that any further increase in the substrate concentration will not produce further increases in the reaction rate, which at saturation depends exclusively on the amount of enzyme. According to the SI system, the unit of the enzymatic activity is the katal, whereby 1 katal is defined as the amount of enzyme that converts 1 mol of substrate (or forms 1 mol of product) in 1 s. However, most frequently, enzymatic activity is expressed in international units (IU); 1 IU is the amount of enzyme that converts 1 μmol of substrate (or forms 1 μmol of product) in 1 min [104]. Enzyme activity is strictly influenced by pH, and almost all enzymes have a pH optimum, at which they show their highest activity. This depends on the protonation of the functional groups of the amino acids and on the three-dimensional structure of the enzyme. The turnover number, *k*_cat_ = *V*_max_/[*E*_T_], can be derived from the enzyme activity, which defines the maximum number of substrate molecules converted to product per unit of time by a single catalytic site. Through the Michaelis-Menten equation, it is also possible to determine the Michaelis constant, *K*_m_, which corresponds to the substrate concentration that results in half saturation of the enzyme, and which represents the affinity of the enzyme for the substrate: the higher the *K*_m_, the lower the enzyme affinity for the substrate.

Differences in the amounts of a key enzyme in genotypes with contrasting metabolite contents can lead to different enzymatic activities that can then be detected through differences in *V*_max_. Here, to unequivocally attribute a different enzyme activity to a different enzyme content, differences in the protein level (e.g., as determined by Western blotting), as well as in the transcript abundance (e.g., determined by expression analysis; see Section 4.3) of its encoding gene should also be detected. In addition, differences in enzyme abundance should have no effect on *k*_cat_, as this parameter is normalized to the amount of enzyme (*E*_T_). When the different metabolite content in contrasting genotypes is due to the presence of different allozymes of a key enzyme, differences in the catalytic parameters are detected that are the consequence of their different amino acid sequences and protein structures. In this case, differences in the *V*_max_ can be observed that are not accompanied by parallel differences in protein and transcript abundances, but rather by differences in *k*_cat_. In addition, a different substrate affinity can be observed when the amino-acid substitutions affect the catalytic site. Amino-acid substitutions at different protein sites can also affect the pH optimum of an enzyme.

### 4.3. Molecular Analysis

Together with enzymatic analysis, allele mining and expression analysis have to be exploited in contrasting genotypes to identify candidate genes that are potentially responsible for variations in a trait. Once a key regulatory enzyme has been identified and characterized, a search is carried out in the public databases to identify its encoding gene. If the genome of the species of interest has been sequenced and annotated, the gene can be searched for using gene annotation information (e.g., GO, Interpro). Alternatively, non-annotated genes can be identified through a BLAST search by comparing them with known genes or proteins from other plant species. Then, the gene can be isolated from the contrasting genotypes by polymerase chain reaction (PCR) strategies and sequenced to identify possible allelic variants. Specific PCRs can be set up to discriminate between different alleles. An InDel polymorphism can be detected by amplifying the DNA region surrounding the polymorphism and comparing the size of the PCR product, whereas a SNP can be detected by using allele-specific PCR (AS-PCR), which includes the competitive allele specific PCR (KASP), the cleaved amplified polymorphic sequences (CAPS) and the pyrosequencing. KASP assay is based on a competitive allele-specific primer and a fluorescence-based reporting system that allow the bi-allelic discrimination of polymorphism [105]. CAPS assays rely on differences in the restriction-enzyme digestion patterns of the PCR fragments between the genotypes caused by a SNP [106]. Pyrosequencing is a sequencing-by-synthesis method that is based on the luminometric detection of the pyrophosphate released upon nucleotide incorporation [107].

In addition to gene-sequence isolation and allele discrimination, PCR can also be used to determine gene expression levels. The discovery of reverse transcriptase led to the development of reversetranscription-PCR (RT-PCR), which is the most widely used method for measuring the number of copies of specific transcripts. Starting with a very small amount of material (usually total RNA), it is possible to copy the RNA by reverse transcription to produce the complementary, single-stranded DNA (cDNA), which is much less susceptible to degradation than RNA. The cDNA can then be amplified by PCR, with a semi-quantitative determination of the amount of transcript obtained by measuring the intensity of the bands of the generated amplicons on agarose gels; in the exponential phase this is directly related to the number of copies of the target cDNA in the sample. To ensure that quantification yields reliable results, normalization is generally achieved by comparing the expression levels of the gene of interest with those of a constitutive control gene that is assumed to have invariant levels of expression. This allows correction for the measured transcript levels in terms of variable starting cDNA amounts and differences in the reverse transcriptase efficiency [108]. RT-PCR is widely used because it is neither difficult nor expensive to perform; moreover, it is characterized by high sequence specificity, high sensitivity, and wide dynamic range of detection.

An important development of RT-PCR was that of real-time quantitative RT-PCR (qRT-PCR; often qPCR), which is based on the use of fluorophore-labeled primers that allow the detection of the amplification products as they accumulate [109]. The main advantage of qRT-PCR over traditional RT-PCR assays is that the starting cDNA amount can be determined quantitatively. Moreover, the data are analyzed in a closed-tube system, thus eliminating the need for post-amplification manipulation. Quantification is most commonly achieved using internal control genes (reference genes), which help to reduce possible errors generated by comparisons between the expression levels of the gene of interest and those of the constitutive control gene [110].

The advent of microarray technology enabled SNP detection and gene expression analysis to be expanded from investigations of a few genes to wide-scale analyses, in which the analysis of many genes can be monitored simultaneously. Different microarrays are commercially available; in particular, microarrays manufactured by Affimetrix are the most commonly used for plant studies [111]. Measurement of transcript levels is based on the hybridization events on a flat surface to which specific probes (i.e., cDNA sequences or oligonucleotides) that are complementary to the transcripts of interest have been fixed and immobilized. For SNP detection, the probes are oligonucleotides that distinguish between different alleles. Microarray technology enables large-scale analyses, although it can have some limitations compared to PCR, as it has lower specificity due to cross-hybridization events, and lower dynamic range of detection.

Alternatively, tag-based sequencing methods have been developed, which include serial analysis of gene expression (SAGE), cap analysis of gene expression (CAGE), and massively parallel signature sequencing (MPSS) [112]. Compared to PCR and microarray, tag-based sequencing approaches have the advantage of not depending on genome annotation for probe selection, although they are based on the more expensive Sanger sequencing technology, and isoforms are generally not distinguishable from each other. These disadvantages have been overcome by the recently defined NGS-based sequencing technologies, such as the Illumina IG, the Applied Biosystems SOLiD, and the Roche 454 Life Science. These platforms can provide information about gene expression, allele-specific expression, novel transcripts, alternative splice sites, and SNPs [113].

These high-throughput approaches are expected to facilitate molecular analysis and expression of genes. However, they are not likely to replace the current RT-PCR, qRT-PCR, and AS-PCR methods, which remain the first choices due to their high sensitivities and specificities, and their low cost. This is particularly the case for researchers who are not equipped for, or do not have expertise to perform, high-throughput analyses, or who are specifically interested in low-throughput applications, which might include detection and quantification of the expression profiles of a few selected genes, such as those that encode enzymes involved in a specific metabolic pathway. Anyway, as indicated above, data obtained using high-throughput gene expression platforms are generally stored in publicly accessible databases, and their consultation has the potential to accelerate candidate gene discoveries.

## 5. Genetics Approaches to Improve Phytochemical Contents in Crops

Marker-assisted selection and genetic transformation are the major tools of the molecular breeding strategies of today. Genome editing is a new technology that promises to contribute greatly to future crop improvements. The choice of one or the other method depends strictly on the genetic variation of the desired trait in the genetic material that is available. MAS is used when the genes/alleles required for the development of new, superior genotypes are already present within the gene pool of the species of interest or in a sexually compatible species. Genetic transformation and genome editing can be used both to introduce native genes/alleles from the gene pool of the crop itself, and to extend the gene pool of the recipient species by introducing genes from non-sexually compatible species. The main advantage of the genetic transformation and genome editing approaches compared to MAS is that they can achieve the same alterations much quicker. Moreover, these advanced approaches allow the targeted improvement of a desired trait without altering the genetic constitution of the recipient genotype, as they do not transfer unwanted genetic material along with the desired gene or allele (i.e., linkage drag).

### 5.1. Marker-Assisted Selection

A DNA marker is a small region of DNA that is tightly linked to the gene of interest and that reveals a mutation in the gene; this can therefore be used to detect polymorphisms between different individuals in a population [114]. Most polymorphisms are in close proximity to the gene of interest, although as indicated above, the polymorphism can also reside within the gene itself, thus generating different alleles at the same locus. As the reliability of the DNA marker to predict the phenotype increases as its distance from the target gene decreases, the use of the gene itself as the DNA marker enables 100% reliable prediction. As indicated above, sequence polymorphisms between different alleles can be due to InDels, or more frequently, to SNPs. Due to their high abundance in the genome, their co-dominant inheritance, and the low cost of their assay, SNPs represent a powerful tool for application to MAS. It is possible to exploit a SNP using AS-PCR, and thus to specifically amplify the allele that controls the trait. In this way, the polymorphism can be converted into a genetic marker that can be easily assayed. The advantages of MAS over conventional breeding are as follows: (i) genotype screening is more objective and often simpler than phenotype screening; (ii) selection is carried out at the early stages of plant growth, thus significantly reducing the number of lines to be tested in the stages that follow; (iii) selection can be made on a single plant basis, rather than on plant families or plots, as necessary for phenotype selection; and (iv) homozygous and heterozygous plants can be distinguished, thus allowing recessive genes to be maintained without the need for progeny tests for each generation.

With regard to the use of MAS in breeding programs that are aimed at improving the phytochemical content in edible crops, most of the applications relate to increases in carotenoid levels in cereal grains. In particular, in maize, PCR-based markers have been developed for the selection of favorable alleles of *lycopene epsilon cyclase* (*lcyE*), which alters the flux from the α-carotene versus β-carotene branch of the pathway, and *carotene* β*-hydroxylase 1* (*crtB1*), which is responsible for β-carotene hydroxylation [115,116]. Four natural polymorphisms of the *lcyE* gene were found to explain more than half of the variation in the two branches, and a three-fold difference in the levels of provitamin A compounds [115]. For the *crtB1* gene, the selection of the most favorable allele in maize elite inbred parents characterized by low kernel β-carotene (at 1.4 mg/g) resulted in a 12-fold increase in β-carotene content in the *crtB1*-introgressed inbreds. In addition, the reconstituted hybrids developed from improved parental inbreds also showed enhanced kernel β-carotene content, compared to the original hybrids (21.7 vs. 2.6 mg/g) [116]. This increase is due to the reduction in transcript expression of the *crtRB1* allele, which decreases the hydroxylation of β-carotene [117]. Allele mining and marker development are also underway for other genes of the carotenoid biosynthetic pathway in maize, including those that encode phytoene synthase and carotenoid cleavage dioxygenases [118].

Promising findings have also been obtained in the identification of genes suitable for MAS of fruit with anthocyanin accumulation. In particular, allele mining has been carried out in apple for the gene that encodes the transcription factor MdMYB1, which regulates anthocyanin accumulation in the fruit peel. The results obtained have highlighted the presence of the *MdMYB1-1* allele that is associated with the red peel color of the fruit, and a CAPS marker was developed for this allele [119]. This was then successfully tested in different segregating populations, in which it provided approximately 80% predictability [120]. A potentially useful marker has also been developed from the InDel in the *MdMYB10* gene, which is responsible for anthocyanin accumulation in the fruit flesh, which is only present in the red fruit core genotypes [121].

### 5.2. Genetic Transformation

Genetic transformation is a process by which a crop species is altered by the incorporation into its genome of foreign genes and regulatory elements. These genetic elements can be manipulated before they are combined, and transferred into a new background, to produce a so-called genetically modified organisms. Only one or a few genes can be transferred by any single transformation event; moreover, neither the number of copies nor the position of the foreign gene in the recipient genome can be controlled. Genes can be moved between any species. Cisgenesis provides for the transformation of a crop species with genes isolated from cross-compatible species, which also include their own introns and promoter and terminator regions, in a sense orientation. In intragenesis, the coding regions and the regulatory elements derive from different genes of a cross-compatible species that have been differently combined. In transgenesis, the genes and their regulatory elements are transferred between non-crossable crop species.

At present, there are two main plant transformation methods: the *Agrobacterium*-mediated method, and particle bombardment. The *Agrobacterium*-mediated method uses *Agrobacteriun tumefaciens* to deliver the foreign gene into the host genome. In nature, this soil bacterium causes tumor formation (i.e., crown galls) on a large number of dicotyledonous species, as well as on some monocotyledonous species and gymnosperms, by transferring a specific DNA fragment (the transferred DNA; T-DNA) from its tumor-inducing plasmid to the plant cells [122]. Recombinant *Agrobacterium* strains in which the native T-DNA is removed and replaced with the gene of interest are thus used for the introduction of foreign genes into plants. However, monocotyledons, and in particular cereal crops, are not susceptible to *Agrobacterium*, and therefore they cannot be transformed using this method. Particle bombardment can overcome the host-dependency of the *Agrobacterium*-mediated method. This technique provides for the delivery of the genetic material using highly accelerated gold or tungsten particles, such that they can penetrate through the cell wall. The foreign DNA coated on their surface will then be freed and will recombine with the chromosomal DNA of the target cells [123].

Genetic transformation has been mainly used to improve staple crops with respect to their carotenoid and flavonoid contents. As for carotenoids, enrichment of β-carotene has been achieved in rice, potato, white maize, and cassava, while enrichment in xanthophylls has been achieved in carrot, tomato, potato, and maize [124]. In particular, the genes that encode the daffodil phytoene synthase (*psy*) and lycopene β-cyclase (*lcy*), together with the gene that encodes bacterial phytoene desaturase (*crtI*), have been transferred to increase the β-carotene content in rice grains to 1.6 μg/g, thus generating the so-called “Golden rice” [125]. Successively, a “Golden Rice 2” was developed by introducing a more efficient maize *phytoene synthase* gene in combination with the bacterial *crtI* used to generate the original “Golden Rice”, thus obtaining an increase in the total carotenoids, as mainly β-carotene, of up to 23-fold, compared to the original “Golden Rice” [126]. Another example is carotenoid improvement in tubers of potatoes transformed with a mini-pathway of bacterial origin, which has given rise to “Golden potatoes”, which have an approximately 20-fold increase in their total carotenoids, and a 3600-fold increase in β-carotene [127]. Alternatively, an increase in carotenoids (of up to 10-fold) has been obtained in tubers by transforming potato plants with the *Or* gene, which encodes a DnaJ cysteine-rich-domain-containing protein that can trigger differentiation of proplastids and/or non-colored plastids into chromoplasts, thus providing a metabolic sink for carotenoid accumulation [128].

For flavonoids, enrichment by transformation has been achieved mainly in tomato, using different approaches. For instance, an up to 78-fold increase in total flavonols in tomato peel was obtained through ectopic expression of petunia *chalcone isomerase*, while in the fruit flesh, the concomitant expression of the sequences that encode chalcone synthase and flavonol synthase from petunia induced increases in flavonol accumulation [129]. Alternatively, ectopic expression of two maize transcription factors, Lc and C1, together with the biosynthetic gene *chalcone isomerase*, resulted in a similar phenotype [129]. Transgenic tomato fruit were also obtained by overexpression of a grapevine gene that encodes the enzyme stilbene synthase, which provides accumulation in the tomato peel of stilbenes, and particularly resveratrol, and other naturally occurring polyphenols [130].

### 5.3. Genome Editing

Site-specific DNA editing using engineered endonucleases has rapidly become a powerful tool for genome editing in diverse eukaryotic organisms, including plants ([131] and references therein). The widespread application of this technology has been largely fueled by the development of the clustered regularly interspaced short palindromic repeats (CRISPR)-associated 9 (Cas9) endonuclease system [132]. CRISPR-Cas are immune systems in bacteria and archaea for protection against foreign nucleic acids [133]. Type II CRISPR-Cas systems contain short direct repeats that are interspaced at regular intervals by sequences incorporated from foreign DNA (protospacers). Transcripts from CRISPRs are processed into short CRISPR RNAs (crRNAs), with each including a protospacer and part of the CRISPR repeat. This crRNA hybridizes with a trans-activating crRNA (tracrRNA) to form a crRNA:tracrRNA duplex, which is, in turn, complexed to Cas9. The protospacer portion of the crRNA guides Cas9 to cleave the complementary foreign DNA sequence if this is adjacent to a short sequence known as a protospacer-adjacent motif.

This system has been adapted to easily and rapidly induce modifications to endogenous genes [132]. In the simplest form of CRISPR-Cas9 technology, the eukaryotic cell is transfected to heterologously express Cas9 and a single guide RNA (sgRNA); this latter consists of a synthetic fusion of crRNA and tracrRNA that is designed to direct Cas9 to cleave a complementary 20-bp sequence that is flanked by the NGG protospacer-adjacent motif sequence. The Cas9-induced double-strand break is repaired via non-homologous end joining. As this non-homologous end joining is an error-prone repair, small insertions and/or deletions can be introduced into the double-strand break, which interrupts the frame, and inactivates the gene. A more precise repair can be achieved by inducing homology-directed repair. With this strategy, the double-strand break is repaired using a donor DNA template that has homology to sequences that flank the target DNA. This enables specific mutations to be introduced, thus generating favorable alleles associated with traits of interest.

Genome editing using the CRISPR-Cas9 system is a new technology, but its use in the genetic improvement of food crops appears to be very promising. Indeed, the first studies have demonstrated that the CRISPR/Cas9 system is highly efficient in rice for inducing target gene editing, with several genes with diverse functions being tested, including *phytoene desaturase* (*OsPDS*), which is important in carotenoid biosynthesis. Homozygotes of the edited target genes were readily found in the T0 plants, and the gene mutations were passed on to the next generation following classic Mendelian law, without any detectable new mutation or reversion [134]. Similarly, the CRISPR/Cas9 system has been used successfully for targeted modification of the *CsPDS* gene into sweet orange [135]; also in this case, no off-target mutations were observed.

## 6. A Case Study: The *Lipoxygenase-B1* (*Lpx-B1*) Genes and the Regulation of the Final Carotenoid Content in Pasta Products

A yellow-amber color represents an important parameter in the assessment of pasta quality. This is due to the presence in the durum wheat endosperm of the carotenoid pigments, which are mainly represented by lutein. Therefore, as well as its role as an esthetic parameter that is strongly appreciated by consumers (who prefer a yellow to amber color, rather than a brown color), the lutein content also represents an important nutritional parameter. Indeed, several findings support the association between dietary intake of foods rich in lutein and lower incidence of degenerative diseases, such as macular degeneration and cancers (see Section 3). Pasta products have a large *per capita* consumption, and they can help in the meeting of the daily recommended dose of lutein (which is still too low) better than other foods. Therefore, genetic improvement of the carotenoid content in pasta products represents a valuable goal to be pursued. 

Different analytical procedures have been developed to determine the amounts of carotenoid pigments in durum wheat grain. Sensitive, selective, and accurate determination has been achieved through HPLC techniques. This chromatographic separation can be achieved using either normal or reverse-phase HPLC after extraction of the carotenoids with the appropriate organic solvents (e.g., ethanol, butylated hydroxytoluene, methanol/tetrahydrofuran) [136,137,138,139,140]. This approach was used by Digesù and coworkers [139] to screen a large durum wheat germplasm collection for carotenoid content in the endosperm. Great variability of this trait has been observed in this crop, with a total carotenoid concentration that ranges from 1.178 to 4.416 μg/g.

However, although it is sensitive and accurate, HPLC analysis is costly and time-consuming, and is therefore not suited for large-scale screening. For this reason, other methods based on light spectrophotometry and reflectance measurements have been developed. These methods do not give information about the composition of the carotenoids, but give overall information on the total yellow pigment content. A colorimetric method for determination of the yellow pigment concentration (YPC) has been developed that is based on extraction in water-saturated *n*-butanol and subsequent spectrophotometric measurement of the absorbance of the alcoholic extract at 435.8 nm [141]. Recently, this method was revised and improved to allow carotenoid content measurements for a single seed, and thus to make the screening of large populations easier during breeding programs when the material available is limited [142,143]. Reflectance measurements are obtained using a chromameter (Minolta CR-300; Konica Minolta Pty Ltd., Macquarie Park, NSW, Australia) equipped with a pulsed xenon arc lamp. This provides absolute measures of the L* (lightness), a* (red-green chromaticity), and b* (yellow-blue chromaticity) coordinates of the Munsell color system, using D65 lightning [144]. The b* value represents the variation in the yellow intensity, and it is also known as the Yellow index (YI). NIR spectroscopy is also used for yellow color measurements for durum wheat grain. However, although this technique is rapid, non-destructive and cost-effective, the instrument calibration requires a very large sample set, and for this reason it has not been greatly used in durum wheat breeding [145,146]. Conversely, both YPC and YI represent good tests for assessing grain and semolina color, as they are highly correlated with the total carotenoid concentration determined by HPLC analysis (0.94, *p* ≤ 0.001; 0.88, *p* ≤ 0.001; respectively) [139]. Consistent with this, and in line with HPLC measurements, large variability has also been observed in durum wheat grain for both YPC (from 3.7 to 12.3 μg/g) [139,147] and YI (from 12.0 to 19.1) [139]. Interestingly, regardless of the method used, modern commercial genotypes show significantly higher carotenoid content compared to the older genotypes [139,147]. These findings confirm that semolina color is a trait worthy of attention, and attests to the focus of the breeders to improve it.

**Figure 6:**
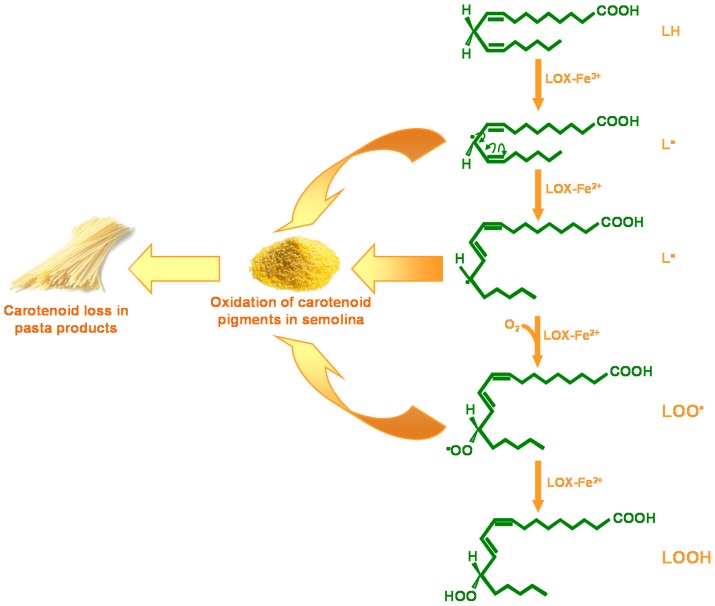
Reaction catalyzed by lipoxygenase (LOX) during pasta processing. LOX catalyzes hydroperoxidation of free linoleate (LH), to give the corresponding hydroperoxide (LOOH). During pasta processing the pentadienyl (L^•^) and peroxy radicals (LOO^•^) of linoleate oxidize the semolina carotenoid pigments, thus leading to carotenoid loss and discoloration of the end product.

However, high carotenoid content in the endosperm does not guarantee an equally high content in pasta products. This is because during pasta processing, oxidative degradation of carotenoids can occur, which leads to carotenoid loss and discoloration (bleaching) of the end product. The main enzymes responsible for this degradative phenomenon are the lipoxygenases (LOX, linoleate:oxygen oxidoreductase; EC 1.13.11.12), which are a class of non-heme iron-containing dioxygenases that catalyze the positional and specific dioxygenation of polyunsaturated fatty acids that contain one or more 1,4-*cis*, *cis*-pentadiene structures (e.g., linoleic, linolenic, arachidonic acids), to give the corresponding hydroperoxide (Figure 6). The radicals produced in the intermediate steps of this reaction can cause oxidation of carotenoid pigments in semolina, and consequently, pigment loss in the pasta products.

Both the LOX-catalyzed linoleate hydroperoxidation and carotenoid bleaching reactions can be monitored spectrophotometrically. Linoleate hydroperoxidation activity can be determined by using this fatty acid as the substrate, and following the absorbance increase at 234 nm [148]. This is due to conversion of linoleate into the corresponding hydroperoxide, which has a conjugated diene with maximum absorbance at this wavelength. The bleaching activity is determined in the presence of linoleate and β-carotene as substrates, by monitoring the absorbance decrease at 460 nm that is due to oxidation of β-carotene [148]. In durum wheat semolina, there is large variability with respect to the LOX activity (see below). This suggests that the durum wheat germplasm has different genes/alleles that contribute to the natural variation of this trait. Among these, the favorable gene/allele is the one associated with very low LOX activity in durum wheat endosperm. As described below, this has been identified and is now used to select superior genotypes in which the carotenoid loss during pasta processing is strongly reduced, thus ensuring high carotenoid levels in the end products.

In cereals, the *LOX* genes have been isolated from different species, including rice [149,150], maize [151,152], barley [153,154,155], common wheat [156,157,158,159], and durum wheat [160,161,162]. In particular, the existence of different LOXs has been intensively investigated in barley, which is a species closely related to durum wheat. Three cDNA sequences, *LoxA*, *LoxB*, and *LoxC*, that encode the LOX proteins have been isolated from barley grains [153,154,155]. The *LoxA* and *LoxC* genes are located on chromosome 4HS and encode, respectively, the LOX-1 and LOX-2 isoforms [163], whereas *LoxB* is located on chromosome 5HL and encodes a LOX isoform that has not been identified to date. LOX-1 is the predominant isoform in mature and quiescent grains, in which it accounts for most of the total LOX activity [154,155].

In durum wheat, the *LOX* genes are located in regions that are collinear with barley: two *Lpx-2* sequences orthologous to the barley *LoxC* gene have been identified on the group 5 chromosomes, and two *Lpx-3* sequences orthologous to the barley *LoxB* gene have been mapped to the group 4 chromosomes [160]. For the durum wheat genes that are orthologous to barley *LoxA*, a partially deleted copy of *Lpx-1* (*Lpx-A1_like*) has been found on the A genome [158,159,160,161], whereas five *Lpx-1* sequences have been identified on the short arm of chromosome 4B [162]. Three sequences correspond to different alleles of the *Lpx-B1.1* gene, referred to as *Lpx-B1.1a*, *Lpx-B1.1b*, and *Lpx-B1.1c*, while the other two sequences correspond to the *Lpx-B1.2* and *Lpx-B1.3* genes. There is also evidence that in durum wheat there is a major QTL that has its peak at the *Lpx-B1* locus; this is associated with most of the LOX activity in semolina [164] and explains a large portion of the variation in pasta color [165]. Similarly, in common wheat, a QTL for LOX activity has been mapped to chromosome 4BS [166,167]. These findings are in line with previous observations on barley LOXs, and they strongly suggest that, in durum wheat, LOXs-1 encoded by genes/alleles at the *Lpx-B1* locus contribute almost exclusively to the total activity detected in semolina, and thus these have a major role in the oxidation of carotenoid pigments that occurs during pasta processing.

The *Lpx-B1* genes and alleles share a common structure, which comprises six exons and seven introns [162]. It is of note that there is a length polymorphism in the last intron of the *Lpx-B1* genes and alleles, due to the presence of a miniature inverted-repeat transposable element (MITE) of the *Stowaway* class [162,163,164]. This MITE is completely absent in the *Lpx-B1.2* and *Lpx-B1.3* genes, whereas, for the *Lpx-B1.1* gene, there is a complete MITE in the *Lpx-B1.1a* allele that is followed by a partial MITE, and in the *Lpx-B1.1b* allele, there is a partially deleted MITE; in contrast, the *Lpx-B1.1c* allele has a large deletion that covers the region comprised between the second intron and the first part of the last exon [162,163,164]. These differences are probably due to different insertions/deletions events, as well as large-scale rearrangements, that have affected the *Stowaway*-type MITE and its adjacent sequences, and that in the *Lpx-B1.1c* allele have caused a large deletion, with the consequent loss of function. Based on the distribution of the *Lpx-B1* genes and alleles in the durum wheat germplasm, three different groups of genotypes can be distinguished: haplotype I, which carries the *Lpx-B1.1b* allele associated with the *Lpx-B1.3* gene; and haplotypes II and III that comprise the genotypes that carry the *Lpx-B1.1a* and *Lpx-B1.1c* alleles, respectively, associated with the *Lpx-B1.2* gene [162] (Table 4). As a consequence, haplotypes I and II have two functional LOX-1 isoforms, whereas there is only one functional LOX-1 isoform in haplotype III. Interestingly, only those genotypes released before 1970 included all of these three haplotypes, whereas the genotypes released in the following years included only haplotypes II and III [162] (Table 4). The *Lpx-B1* locus maps in the short arm of the chromosome 4B, near the *Rht-B1b* dwarfing gene [160,162,164], which was introgressed in durum wheat in the 1970s from the common wheat cultivar ”Norin 10” [168]. Therefore, the distribution of the *Lpx-B1* genes and alleles in durum wheat germplasm could reflect the selection carried out on the *RhtB1b* locus from the 1970s onwards.

The *Lpx-B1* genes and alleles differ in their expression levels. All three of the *Lpx-B1.1* alleles and the *Lpx-B1.3* gene are expressed at high levels, whereas the *Lpx-B1.2* gene is expressed at very low levels [162] (Table 4). As a consequence, and taking into consideration only those transcripts that encode functional LOXs, haplotype I is characterized by the highest overall transcript levels, with intermediate and very low expression levels for haplotypes II and III, respectively. This trend is reflected in the LOX activity of the three haplotypes. Indeed, genotypes belonging to haplotype I have the highest LOX activity, when monitored both as linoleate hydroperoxidation and β-carotene bleaching; further, genotypes of haplotype II are characterized by intermediate LOX activity, while haplotype III is characterized by low and very low activity [147,162] (Table 4).

**Figure 7:**
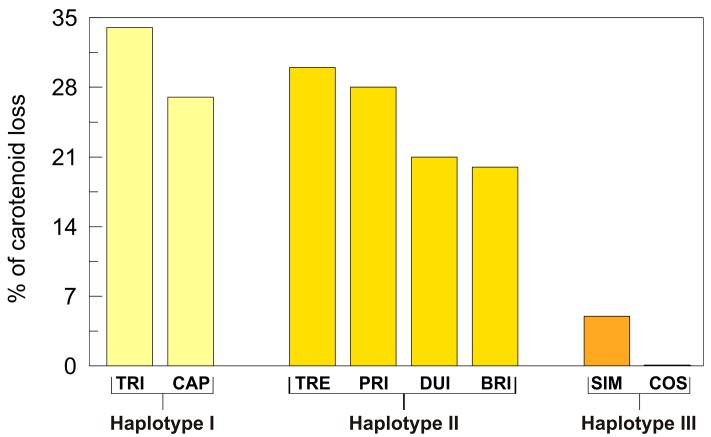
Carotenoid loss during pasta processing. Cultivars: TRI, Trinakria; CAP, Capeiti 8; TRE, Tresor; PRI, Primadur; BRI, Brindur; SIM, Simeto; COS, Cosmodur. Data from [169].

In line with both the molecular and biochemical findings, the loss of carotenoids that occurs during pasta processing is very low in haplotype III genotypes, which have the deleted *Lpx-B1.1c* allele and have very low LOX activity in the endosperm (Figure 7) [169].

Although the high LOX activity haplotype I has disappeared as a consequence of the selection for the short straw, there remains large variability in LOX activity in the modern genotypes, with values ranging from 4.22 to 8.97 IU/g semolina [147,162]. Therefore, the selection of haplotype III, which has the deleted *Lpx-B1.1c* allele, might be a useful tool to fix low LOX activity in the semolina of all new genotypes, consequently providing high carotenoid content in the end products. Consistent with this, simple PCR-based marker systems have been set up to test the presence/absence of the deletion and differentiate *Lpx-B1* alleles in MAS programs. For instance, deletion associated at the *Lpx-B1.1c* allele has been included in the list of markers used in the WheatCAP project (a consortium of more than 20 US breeders), the main objective of which is to use modern selection technologies to increase the competitiveness of public wheat breeding programs [170]. Currently, this marker is included in a MAS program that is aimed at the pyramiding of the genes responsible for disease resistance and qualitative traits into an elite cultivar of durum wheat (PR22D89) [171,172,173].

## 7. Conclusions

More and more evidence has become available that regular consumption of fruit, vegetables, and whole grains is associated with reduced risk of developing chronic diseases, and that the health benefits of plant-based foods are due to their phytochemical content. However, there is a huge gap between the average intake of these compounds and the recommended doses. For this reason there is strong interest in the nutritional improvement of this plant-based food supply. The combination of the new tools for metabolic and gene expression analyses with classical techniques of enzymatic analysis can be put in use to address the functional identification of the key genes in plant genomes that regulate the accumulation of a desired phytochemical. Although, to date, the whole genome sequence is available for a limited number of plant species, the recently emerged cheap and massive DNA sequencing technologies can be exploited to meet the challenge of genome sequencing also in non-model plants. Together with integration of the increasing databases relating to transcript, metabolite and protein levels, this will allow the decoding of the gene functions, and thus the unravelling of the causal links between specific molecular traits and the level of health-promoting compounds in crops. These insights will aid breeders in the formulation of advanced improvement approaches that are more closely targeted to nutrition-related issues.

## Figures and Tables

**Table 1:** The main classes of phenolic compounds according to their carbon chains.

Class	Carbon Skeleton	Examples
C_6_	Simple phenols and benzoquinones	Catechol, 2,6-dimethoxybenzoquinone, hydroquinone, phenol, pirocatechol
C_6_–C_1_	Benzoic acids	Gallic, *p*-hydroxybenzoic, salicylic, siringic and vanillic acids
C_6_–C_2_	Phenylacetic acids and acetophenones	3-Acetyl-6-methoxybenzaldehyde, tyrosol, *p*-hydroxyphenylacetic acid
C_6_–C_3_	Cinnammic acids and phenylpropanoids	Caffeic, ferulic, *p*-coumaric, sinapic acids, aesculetin, bergenon, eugenin, myristicin
C_6_–C_4_	Naphtochinones	Juglone, plumbagin
C_6_–C_1_–C_6_	Xanthones	Mangiferin, mangostin
C_6_–C_2_–C_6_	Stilbenes and anthraquinones	Astringin, resveratrol, viniferin, chrysophanol, emodin
C_6_–C_3_–C_6_	Flavonoids and isoflavonoids	Catechin, cyanidin, epigallocatechin-3-gallate, naringenin, quercetin, myricetin, genistein
(C_6_–C_3_)_2_	Lignans and neolignans	Lariciresinol, pinoresinol, eusiderin, magnolianin
(C_6_)*_n_*	Catechol melanins	
(C_6_–C_1_)*_n_*	Hydrolysable tannins	
(C_6_–C_2_)*_n_*	Lignin, cutin and suberin	
(C_6_–C_3_–C_6_)*_n_*	Condensed tannins (proanthocyanidins)	

**Table 2:** Classification of terpenes and terpenoids on the basis of their isoprene units.

Class	Isoprene Units	Carbon Atoms	Examples
Monoterpene	2	10	Canphor, eucalyptol, geraniol, lavandulol, limonene, menthol, pinene, thymol
Sesquiterpene	3	15	Abscisic acid, bergamotene, cedrol, curcumene, patchoulol, vetivone
Diterpene	4	20	Abietic acid, cafestol, caffeol, carnosol, gibberellin, phytol
Triterpene	6	30	Betulinic acid, morolic acid, oleanolic acid, ursolic acid, brassinosteroids, saponins
Tetraterpene	8	40	Carotenoids (e.g., α-carotene, β-carotene, crocin, licopene) and xanthophylls (e.g., lutein, zeaxanthin, α-cryptoxanthin, β-cryptoxanthin)
Polyterpene	>8	>40	Cytokinines, gutta-percha, rubber

**Table 3:** Classification of glucosinolates on the basis of their amino-acid precursor.

Class	Example	Structure
Aliphatic	Sinigrin	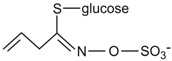
	Glucoraphanin	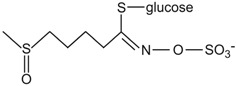
	Dehydroerucin	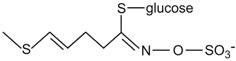
Indole	Glucobrassicin	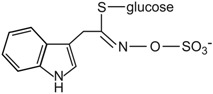
Aromatic	Sinalbin	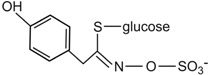
	Gluconasturtiin	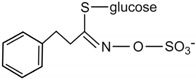

**Table 4:** Molecular and biochemical characteristics of the three *Lpx-B1* haplotypes in durum wheat germplasm.

Haplotype ^a^	Year of Release	Gene/Allele ^a^	Transcript Abundance ^a^	Linoleate Hydroperoxidation Activity (IU/g) ^a,b^	β-Carotene Bleaching Activity (IU/g) ^a,b^
I	Pre–1971	*Lpx-B1.1b* *Lpx-B1.3*	High High	2.30–7.91 (4.60)	0.074–0.037 (0.058)
II	1971–1990	*Lpx-B1.1a* *Lpx-B1.2*	High Very low	1.32–4.52 (2.59)	0.026–0.043 (0.034)
III	1991–2005	*Lpx-B1.1c* *Lpx-B1.2*	High Very low	0.22–0.02 (0.12)	0.007–0.003 (0.005)

^a^ Data from [162]; ^b^ Means are given in brackets.

## Abbreviations

AD	Alzheimer’s disease
AS-PCR	Allele specific PCR
CAGE	Cap analysis of gene expression
CAPS	Cleaved amplified polymorphic sequences
CRISPR	Clustered regularly interspaced short palindromic repeats
crRNA	CRISPR RNA
CVD	Cardiovascular diseases
GC	Gas chromatography
HPLC	High-performance liquid chromatography
InDel	Insertion/deletion
IPP	Isopentenyl pyrophosphate
KASP	Kompetitive allele specific PCR
LC	Liquid chromatography
LOX	Lipoxygense
*Lpx-B1*	*Lipoxygenase-B1*
MAS	Marker-assisted selection
MITE	Miniature inverted-repeat transposable element
MPSS	Massively parallel signature sequencing
MS	Mass spectrometry
NGS	Next-generation sequencing
NIR	Near-infrared
NMR	Nuclear magnetic resonance
PCR	Polymerase chain reaction
RT-PCR	Reverse transcription-PCR
qRT-PCR	Real time quantitative RT-PCR
QTL	Quantitative trait locus
SAGE	Serial analysis of gene expression
sgRNA	Single guide RNA
SNP	Single nucleotide polymorphism
T-DNA	Transferred DNA
tracrRNA	Trans-activating crRNA
YI	Yellow index
YPC	Yellow pigment concentration
